# Is tumor necrosis a clinical prognostic factor in hepato‐biliary‐pancreatic cancers? A systematic review and meta‐analysis

**DOI:** 10.1002/cam4.5742

**Published:** 2023-03-23

**Authors:** Siqi Yang, Haijie Hu, Yafei Hu, Yushi Dai, Ruiqi Zou, Tianrun Lv, Fuyu Li

**Affiliations:** ^1^ Department of Biliary Surgery West China Hospital of Sichuan University Chengdu Sichuan Province China

**Keywords:** hepato‐biliary‐pancreatic cancer, meta‐analysis, prognosis, tumor necrosis

## Abstract

**Background:**

It has been proven that tumor necrosis is associated with poor prognoses in various solid malignant tumors. However, the prognostic effect of tumor necrosis in hepato‐biliary‐pancreatic cancers is still unclear. Therefore, this study was performed to evaluate the associations of tumor necrosis with survival outcomes and clinicopathological features in patients with hepato‐biliary‐pancreatic cancers.

**Methods:**

Based on the PRISMA statement, eligible studies were identified from PubMed, Embase, Cochrane Library, and Web of Science from inception until January 2023. The pooled hazard ratios (HRs) and 95% confidence intervals (95%CIs) were calculated to assess the connection between tumor necrosis and hepato‐biliary‐pancreatic cancers. We then choose which effects model to use to generate pooled HRs and 95% CIs, depending on data heterogeneity.

**Results:**

In total, 6497 articles were identified, 10 of which were included in this meta‐analysis. Our results suggested that the presence of tumor necrosis predicted a poorer outcome for overall survival (HR = 1.54, 95% CI = 1.35–1.77, *p* < 0.001) and recurrence‐free survival (HR = 1.69, 95% CI = 1.37–2.08, *p* < 0.001). In addition, tumor necrosis was correlated with larger tumor size, a higher frequency of lymph node metastasis, poorer histologic differentiation, and higher recurrence and metastasis rates.

**Conclusion:**

Our meta‐analysis suggests that hepato‐biliary‐pancreatic cancer patients with tumor necrosis have dismal survival outcomes, and that their tumors have aggressive biological behaviors. Tumor necrosis has the potential to be a promising biomarker for forecasting poor prognosis in these patients.

## Graphical abstract

Hepato‐biliarypancreatic cancer patients with tumor necrosis have dismal survival outcomes, and their tumors have aggressive biological behaviors. Tumor necrosis has the potential to be a promising biomarker for forecasting poor prognosis in these patients.

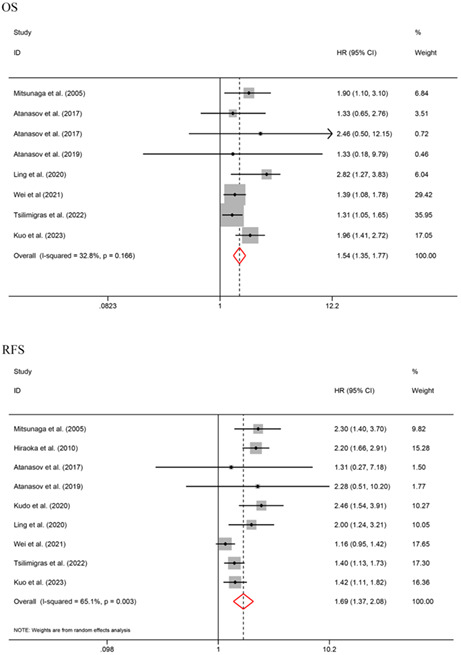

## 1 INTRODUCTION

Hepato‐biliary‐pancreatic (HBP) cancers include liver cancers, biliary tract cancers, and pancreatic cancers. A recent study reported that approximately 1.5 million new cases of HBP cancers occurred, and 1.4 million patients died of HBP cancers in 2020 worldwide, which accounted for 8% of all new cancer cases and 14% of cancer deaths.^1^ The increasing incidence and mortality of HBP cancers lead to a heavy burden on public health and the economy.^2^ Despite advances in therapeutic strategies, patients with HBP cancer still have poor prognoses, and the survival rate has not increased for decades.^3^, ^4^, ^5^, ^6^ For example, the 5‐year overall survival of liver cancer is approximately 37%–65%, and the recurrence rate is higher than 75%.^7^, ^8^ It tends to be helpful to identify patients with poor prognoses and develop appropriate perioperative planning for these sub‐populations. Therefore, it is crucial to discover more prognostic factors to help guide clinical treatment strategies and improve patients' long‐term survivals. Several pathological features such as, tumor burden, tumor histological differentiation/grade, lymph node metastasis, and vascular and neural invasion, have remained predictors of outcomes in HBP cancer patients.^9^, ^10^, ^11^, ^12^, ^13^ Some of these histopathological parameters are included in the American Joint Committee on Cancer eighth edition TNM staging system to predict the prognoses of HBP cancer patients.^14^, ^15^, ^16^ However, clinical outcomes may vary significantly among patients at the same pathological stage, which indicates that there exist other factors which may also impact the prognoses.^17^

Tumor necrosis is a pathological phenomenon often discovered in solid tumors. The degree of hypoxia within the tumor can reveal the level of necrosis. Increased tumor necrosis may contribute to disease progression, treatment resistance, and poor outcomes.^18^ The prognostic value of tumor necrosis has been studied in renal, breast, lung, and colorectal cancer, indicating that the presence of tumor necrosis is related to lower survival rates.^17^

Several recent studies have suggested that tumor necrosis is likely to affect the outcomes of patients in liver, bile tract, and pancreatic cancer. However, there currently existed no studies systematically clarifying the overall prognostic value of tumor necrosis in HBP cancers. Therefore, this systematic review and meta‐analysis was conducted to assess whether the presence of tumor necrosis affects the prognoses of HBP cancer patients. Our study was performed according to the PRISMA guidelines for the reporting of meta‐analyses.^19^

## 2 MATERIALS AND METHODS

### 2.1 Search strategy

Literature was systematically reviewed by searching PubMed, Embase, Cochrane Library, and Web of Science for studies published from inception up until January 2023. The Medical Subject Heading (MeSH) terms and main keywords accepted were as follows: “tumor necrosis”, “necrosis”, “biliary tract neoplasms”, “biliary cancer”, “cholangiocarcinoma”, “extrahepatic cholangiocarcinoma”, “intrahepatic cholangiocarcinoma”, “liver neoplasms”, “liver cancer”, “hepatocellular carcinoma”, “pancreatic neoplasms”, “pancreatic cancer”, “prognosis”, “survival”, “mortality”, “survival analysis”, and their combinations. The detailed retrieval strategies are provided in the Supplementary material. Additional relevant references were identified by manually searching the reference lists of the selected research articles.

### 2.2 Inclusion and exclusion criteria

Studies meeting the following criteria were considered eligible: (1) patients with HBP cancers underwent surgical resection without receiving any previous therapy (neoadjuvant radiotherapy or chemotherapy); (2) tumor necrosis was confirmed histopathologically; (3) studies that reported the prognostic role of tumor necrosis in HBP cancers including overall survival (OS), disease‐specific survival (DSS), and recurrence‐free survival (RFS); (4) the eligible studies provided hazard ratios (HRs) and 95% confidence intervals (CIs). Otherwise, the studies were asked to provide related data to estimate the HR; (5) A score of ≥6 points on the Newcastle–Ottawa Scale (NOS). The exclusion criteria were as follows: (1) patients who received local treatment, neoadjuvant radiotherapy or chemotherapy before surgery; (2) tumor necrosis was not identified pathologically; (3) the associations between tumor necrosis and clinical outcomes were not reported, and the HRs/95%CIs were unavailable; (4) review articles, letters, commentaries, case reports, and conference abstracts; (5) articles not written in English.

### 2.3 Data acquisition and quality assessments

The investigators (author 1–6) independently recorded related information with a standardized form. Information included author, study year, country, study design, sample size, age of patients, sex ratio, cancer type, histology, and survival end point. The quality assessment of cohort researches was assessed by the NOS^20^ (authors 1 and 2). The NOS consisted of three parts: selection (0–4 points), comparability (0–2 points), and outcome assessment (0–3 points). NOS scores ≥6 were considered high‐quality studies.^21^, ^22^

### 2.4 Statistical analysis

Study characteristics and outcome parameters of all included studies were extracted and entered into a Microsoft Excel spreadsheet. The primary outcome was OS. Crude or adjusted HRs with 95% CIs were extracted for survival directly from each included study. If the HR and 95% CI were provided in the form of a Kaplan–Meier survival curve, the HR was estimated with the curve data digitized by the Engauge Digitizer software (version 10.4, Mark Mitchell). The heterogeneity of the enrolled studies was evaluated using the Cochran's Q test and the Higgins I‐squared statistic. A *P* heterogeneity of <0.10 or *I* ^2^ > 50% suggested significant heterogeneity. Sensitivity analysis was used to discover the sources of heterogeneity. When significant heterogeneity was observed, the random effects model was used. Otherwise, the fixed effects model was adopted. In order to assess the heterogeneity and the effects of cancer type and geographic region more precisely, we performed subgroup analyses. All analyses were conducted using Stata 14.0 software (Stata Corp).

## 3 RESULTS

### 3.1 Literature research and characteristics of studies

**FIGURE 1:**
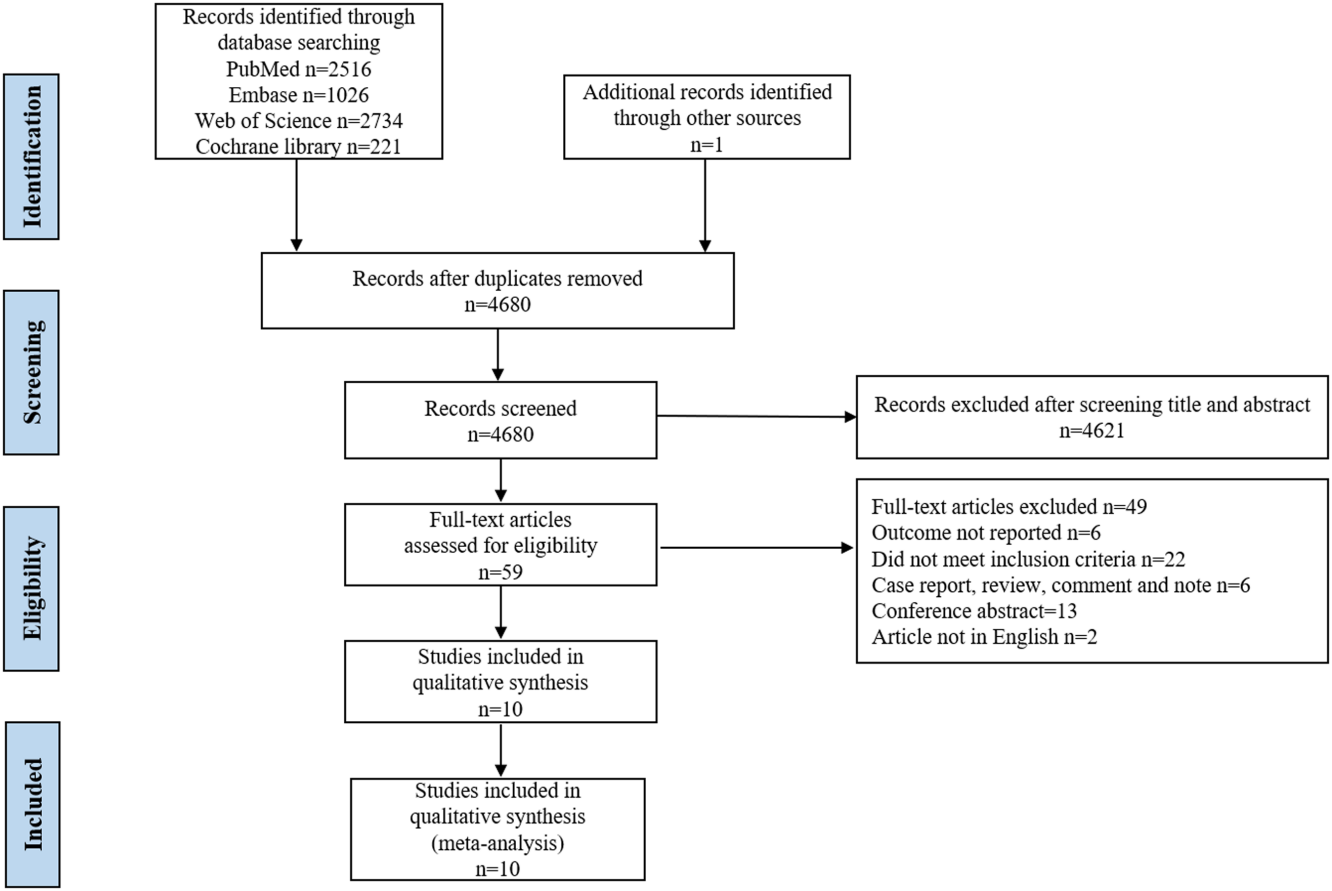
PRISMA flowchart of search strategy and results.

In total, 6392 potentially relevant articles were identified. After excluding 1818 duplicate studies, the titles and abstracts of the 4574 reports were scanned to exclude irrelevant research. In the end, 59 studies were pulled for full‐text evaluation and 10 matching the inclusion criteria were finally included in the meta‐analysis^23^, ^24^, ^25^, ^26^, ^27^, ^28^, ^29^, ^30^, ^31^, ^32^ (Figure 1). The characteristics and details of all included studies are shown in Table 1. The publication years of the included articles were between 2005 and 2023. In total, 3630 patients were included in our meta‐analysis, of which 1668 had tumor necrosis. Of the 10 included studies, three reported on pancreatic cancer (PC), four reported on hepatocellular carcinoma (HCC), two reported on intrahepatic cholangiocarcinoma (ICC), and one reported on hilar cholangiocarcinoma (HC). Further information on each the included studies is detailed in Table S1.

**TABLE 1:** Characteristics and details of the included studies.

Study	Year	Country	Study design	Total population	Median age	Male (%)	Necrosis (+) (n)	Necrosis (−) (n)	Cancer type	Survival analysis
Mitsunaga et al.^21^	2005	Japan	RCS	101	65	56.4	32	69	PDAC	RFS, OS
Hiraoka et al.^22^	2010	Japan	RCS	348	NA	59.2	223	125	PDC	RFS, DSS
Atanasov et al.^23^	2017	German	RCS	88	60	52.3	57	31	ICC	OS
Atanasov et al.^24^	2017	German	RCS	47	NA	NA	28	19	HC	RFS, OS
Atanasov et al.^25^	2019	German	RCS	58	NA	22.4	35	23	HCC	RFS, OS
Kudo et al.^26^	2020	Japan	RCS	221	70	60.2	106	115	PDAC	RFS, DSS
Ling et al.^27^	2020	China	RCS	335	48	88.1	157	178	Solitary small HCC	RFS, OS
Wei et al.^28^	2021	NA	RCS	919	62	80	367	552	HCC	RFS, OS
Tsilimigras et al.^29^	2022	NA	RCS	757	62	48.1	384	373	ICC	RFS, OS
Kuo et al.^30^	2023	China	RCS	756	NA	77.6	279	477	HCC	RFS, OS

Abbreviations: HC, hilar cholangiocarcinoma; HCC, hepatocellular carcinoma; ICC, intrahepatic cholangiocarcinoma; NA, data not available; OS, overall survival; PDAC, pancreatic ductal adenocarcinoma; PDC, pancreatic ductal carcinoma; RCS, retrospective cohort study; RFS, recurrence‐free survival.

### 3.2 Assessment of methodological quality

The Newcastle–Ottawa score of all included studies ranged from 6–8, indicating that all studies were of relatively high quality (Table S2).

### 3.3 Prognostic value of tumor necrosis for survival outcome

**FIGURE 2:**
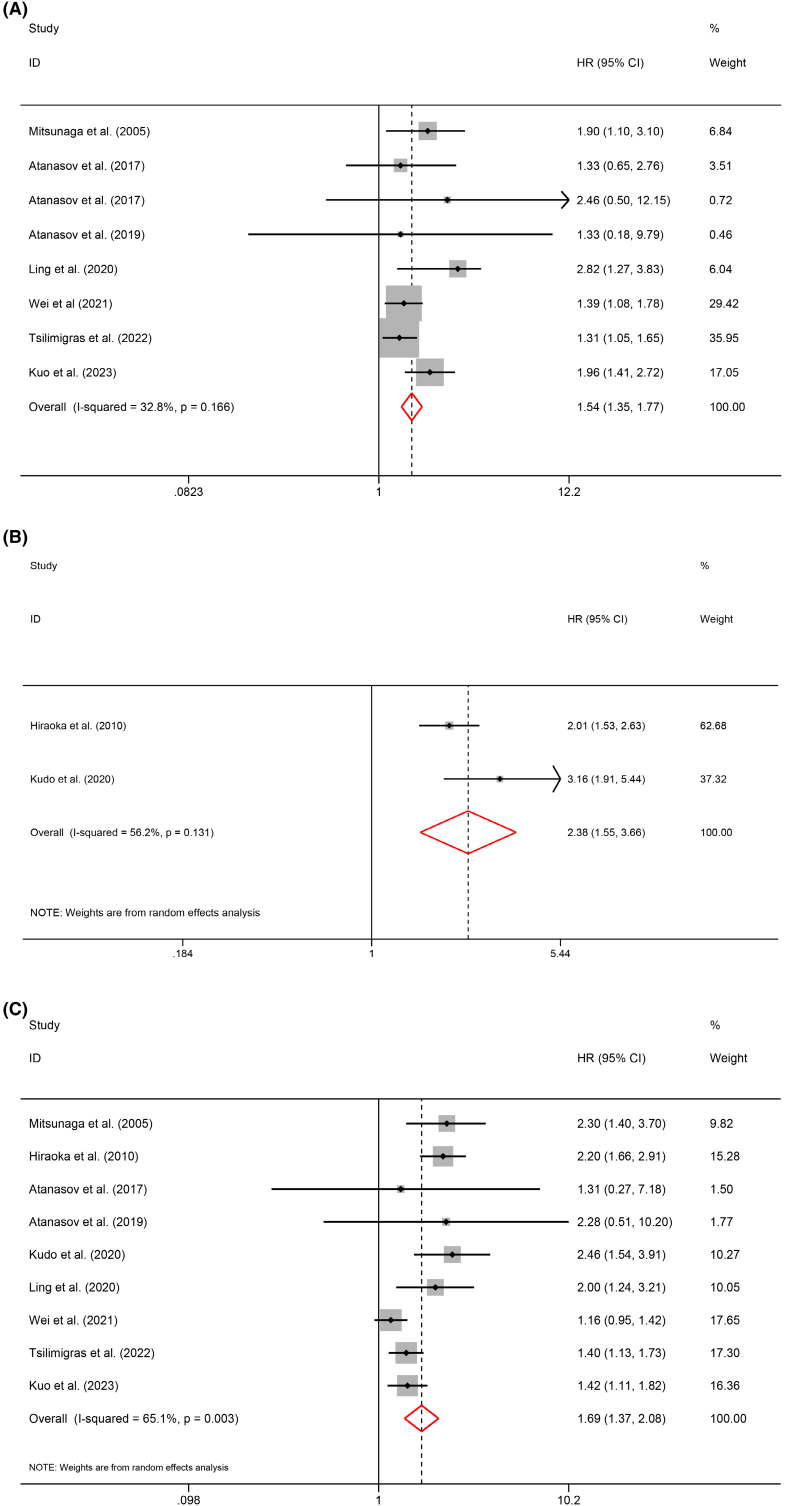
Meta‐analysis of the association between tumor necrosis and survival outcomes of HPB cancers. (A) OS; (B) DSS; (C) RFS

A total of eight articles reported OS, and two studies provided DSS. The meta‐analysis indicated that tumor necrosis in HBP cancers predicted a poor outcome for OS (HR = 1.54, 95% CI 1.35–1.77, *p* < 0.001, *I* ^2^ = 32.8%, *P*_heterogeneity_ = 0.166; fixed‐effects model; Figure 2A) and DSS (HR = 2.38, 95% CI 1.55–3.66, *p* < 0.001, *I* ^2^ = 56.2%, *P*_heterogeneity_ = 0.131; random‐effects model; Figure 2B). The sensitivity analysis result is shown in Figure S1. A total of nine studies reported RFS. The meta‐analysis revealed that HBP cancers presenting with tumor necrosis were associated with worse RFS (HR = 1.69, 95% CI 1.37–2.08, *p* < 0.001, *I* ^2^ = 65.1%, *P*_heterogeneity_ = 0.003; random‐effects model; Figure 2C). The sensitivity analysis failed to discover the source of heterogeneity in the RFS analysis (Figure S2).

Subgroup analysis was performed to evaluate the prognostic value of tumor necrosis for survival outcome in different HBP cancers and for patients from various regions (Figure S3). Owing to the small number of eligible articles, the DSS values^24^, ^28^ of the included studies were considered to be OS values. The results showed that PC had the poorest OS and RFS compared to HCC and cholangiocarcinoma and that HBP‐cancer patients in Asia had the lowest OS and RFS compared to patients in other regions (Table 2). Tumor necrosis may therefore be of higher prognostic value for PC patients and Asia patients.

**TABLE 2:** Subgroup analysis for the cancer type and geographic region.

Analysis	No. of studies	References	Pooled HRs (95% CI)	*p*‐Value	Study heterogeneity		Effects model
					*I* ^2^ (%)	*P*_heterogeneity_	
OS							
Overall	10	[21, 22, 23, 24, 25, 26, 27, 28, 29]	1.81 (1.48, 2.21)	<0.001	53.2	0.023	RE
Cancer type							
Pancreatic cancer	3	[21, 22, 26]^a^	2.15 (1.73, 2.67)	<0.001	21.5	0.280	FE
Hepatocellular carcinoma	4	[23, 27, 28, 30]	1.82 (1.31, 2.54)	<0.001	53.7	0.090	RE
Cholangiocarcinoma	3	[23, 24, 29]	1.33 (1.07, 1.64)	0.010	0.0	0.745	FE
Geographic region							
Asia	5	[21, 22, 26, 27, 30]	2.15 (1.81, 2.56)	<0.001	0.0	0.434	FE
Europe	3	[23, 24, 25]	1.46 (0.78, 2.73)	0.234	0.0	0.785	FE
Multiple regions^b^	2	[28, 29]	1.35 (1.14, 1.59)	0.001	0.0	0.730	FE
RFS							
Overall	9	[21, 22, 24, 25, 26, 27, 28, 29, 30]	1.69 (1.37, 2.08)	<0.001	65.1	0.003	RE
Cancer type							
Pancreatic cancer	3	[21, 22, 26]	2.27 (1.83, 2.82)	<0.001	0.0	0.918	FE
Hepatocellular carcinoma	4	[25, 27, 28, 30]	1.32 (1.14, 1.53)	0.005	44.3	0.146	FE
Cholangiocarcinoma	2	[24, 29]	1.40 (1.13, 1.73)	0.002	0.0	0.937	FE
Geographic region							
Asia	5	[21, 22, 26, 27, 30]	1.96 (1.55, 2.48)	<0.001	51.2	0.085	RE
Europe	2	[24, 25]	1.77 (0.59, 5.36)	0.311	0.0	0.625	FE
Multiple regions^b^	2	[28, 29]	1.27 (1.10, 1.47)	0.011	36.9	0.208	FE

Abbreviations: FE, fixed‐effects; RE, random‐effects.

^a^ The DSS was considered DSS to be equivalent to OS in subgroup analysis.

^b^ The data was obtained from international data base.

**FIGURE 3:**
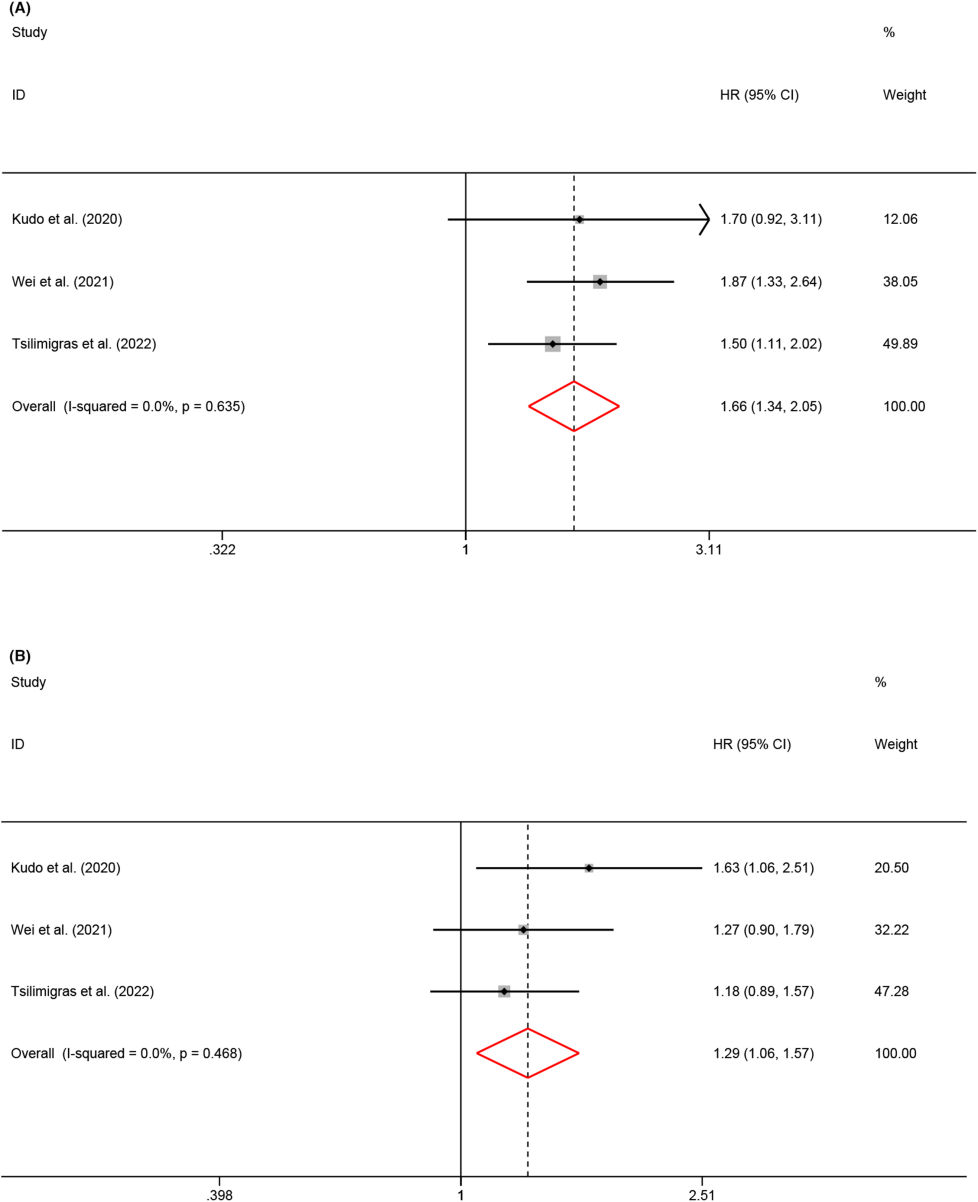
Meta‐analysis of the association between tumor necrosis degree and survival outcomes of HPB cancers. (A) OS; (B) RFS.

We also performed an analysis to clarify whether the tumor necrosis degree impacts the OS and recurrence of HBP cancers. According to the included studies, moderate necrosis and extensive necrosis were defined as a necrosis extent of ≤50% or the maximum diameter of ≤5 mm, and a necrosis extent of> 50% or the maximum diameter of >5 mm, respectively. Our meta‐analysis revealed that extensive necrosis resulted in shorter OS (HR = 1.66, 95% CI 1.34–2.05, *p* < 0.001, *I* ^2^ = 0.0%, *P*_h_ = 0.635; fixed‐effects model; Figure 3A) and RFS (HR = 1.29, 95% CI 1.06–1.57, *p* = 0.01, *I* ^2^ = 0.0%, *P*_h_ = 0.468; fixed‐effects model; Figure 3B).

### 3.4 Association of tumor necrosis with clinicopathological features

We performed meta‐analyses to explore the correlation between tumor necrosis and clinicopathological parameters of patients with HBP cancers, including tumor size, tumor nodule number, lymph node metastases, vascular invasion, neural invasion, pathologic tumor status, histologic differentiation, R0 resection, recurrence, and metastases. In addition, we performed subgroup analysis to assess the associations between different tumors with vascular invasion and pathologic tumor status. The meta‐analysis results revealed that HPB cancer patients who had tumor necrosis were associated with larger tumors (OR = 2.19, 95% CI 1.59–3.01, *p* < 0.001), presence of lymph node metastases (OR = 1.71, 95% CI 1.34–2.18, *p* < 0.001), higher frequencies of vascular (OR = 1.67, 95% CI 1.04–2.70, *p* = 0.035) and neural invasion (OR = 1.54, 95% CI 1.03–2.30, *p* = 0.036), higher tumor status (OR = 1.99 95% CI 1.00–3.96, *p* = 0.049), and poorer histologic differentiation (OR = 1.80, 95% CI 1.50–2.16, *p* < 0.001). Patients with tumor necrosis were more susceptible to recurrence (OR = 1.48 95% CI 1.19–1.84, *p* < 0.001) and metastases (OR = 2.29, 95% CI 1.14–4.60, *p* = 0.020) (Table 3). The subgroup analysis for vascular invasion and tumor status indicated that PC and HCC patients with tumor necrosis tended to present with vascular invasion, and HCC patients generally had higher tumor statuses (Table 3).

**TABLE 3:** Meta‐analysis of the association of tumor necrosis with clinical features.

Analysis	No. of studies	References	Odd ratios (95% CI)	*p*‐Value	Study heterogeneity		Effects model
					*I* ^2^ (%)	*P*_heterogeneity_	
Tumor size^a^	7	[22, 23, 25, 27, 28, 29, 30]	2.19 (1.59, 3.01)	<0.001	71.7	0.002	RE
Multiple tumor nodule	5	[23, 25, 28, 29, 30]	1.12 (0.72, 1.73)	0.615	69.1	0.011	RE
Lymph node metastases	6	[22, 23, 24, 25, 26, 29]	1.71 (1.34, 2.18)	<0.001	44.2	0.110	FE
Neural invasion	3	[22, 23, 26]	1.54 (1.03, 2.30)	0.036	0.0	0.853	FE
Histologic differentiation	6	[22, 23, 27, 28, 29, 30]	1.80 (1.50, 2.16)	<0.001	18.1	0.296	FE
R0 resection	3	[22, 25, 29]	1.19 (0.89, 1.58)	0.244	34.4	0.218	FE
Recurrence	5	[22, 23, 24, 25, 28]	1.48 (1.19, 1.84)	<0.001	1.0	0.401	FE
Metastases	3	[22, 23, 25]	2.29 (1.14, 4.60)	0.020	0.0	0.513	FE
Vascular invasion	8	[22, 23, 25, 26, 27, 28, 29, 30]	1.67 (1.04, 2.70)	0.035	86.4	0.000	RE
Subgroup							
Pancreatic cancer	2	[22, 26]	2.61(1.71, 3.97)	<0.001	0.0	0.456	FE
Cholangiocarcinoma	2	[23, 29]	0.41 (0.10, 1.69)	0.29	51.3	0.152	RE
Hepatocellular carcinoma	4	[25, 27, 28, 30]	2.03 (1.68, 2.46)	<0.001	0.0	0.987	FE
Pathologic tumor status	6	[22, 23, 25, 28, 29, 30]	1.99 (1.00, 3.96)	0.049	86.2	0.000	RE
Subgroup							
Pancreatic cancer	1	[22]	2.28 (0.60, 8.86)	0.225	‐	‐	‐
Cholangiocarcinoma	2	[23, 29]	1.16 (0.41, 3.28)	0.783	77.8	0.034	RE
Hepatocellular carcinoma	3	[25, 28, 30]	2.75 (1.17, 6.48)	0.021	85.1	0.001	RE

Abbreviations: FE, fixed‐effects; RE, random‐effects.

^a^ Classification of tumor size is provided by each included study.

### 3.5 Publication bias

The funnel plot for OS indicated the absence of probable funnel asymmetry. The *p* values of the Begg's test and Egger's test were 0.721 and 0.238, respectively (Figure S14). Therefore, we concluded that no significant publication bias existed in this study.

## 4 DISCUSSION

Many studies have indicated that tumor necrosis has adverse effects on long‐term outcomes in individuals with some cancers including breast, lung thyroid and renal cancer, malignant mesothelioma, and Ewing sarcoma.^18^, ^33^, ^34^, ^35^, ^36^, ^37^ Recently, several meta‐analyses have proven that tumor necrosis has a prognostic impact in some solid tumors, such as in renal cancer and gastrointestinal stromal tumors.^17^, ^21^, ^38^ However, the number of articles in the present study, focusing on the association of tumor necrosis with prognosis of patients with HBP cancers, is small. Furthermore, to our knowledge, there have been no other meta‐analyses published on this topic. As a result, we performed this meta‐analysis to explore the connection between tumor necrosis and clinical consequence of HBP cancers.

Our meta‐analysis enrolled 3630 patients with HBP cancers from 10 studies of which 46.0% of the patients presented with tumor necrosis. Our study showed that necrosis was correlated with worse OS (HR = 1.54, *p* < 0.001), DSS (HR = 2.38, *p* < 0.001), and RFS (HR = 1.69, *p* < 0.001). There was no heterogeneity found in the analyses of the OS model and only slight heterogeneity in the analyses of the DSS and RFS models. We also found that, compared with moderate tumor necrosis, extensive tumor necrosis was a marker for predicting worse OS (HR = 1.66, *p* < 0.001) and RFS (HR = 1.29, *p* = 0.01). Subgroup analysis indicated that PC patients and Asian patients presenting with tumor necrosis had poorer OS (PC: HR = 2.15, *p* < 0.001; Asian patients: HR = 2.15, *p* < 0.001) and RFS (PC: HR = 2.27, *p* < 0.001; Asian patients: HR = 1.96, *p* < 0.001), which suggested that tumor necrosis might play a better prognostic role in PC and Asian HBP cancer patients compared to other HBP cancers and in patients from other regions. Moreover, we found that the presence of tumor necrosis may be correlated with larger tumors, more frequent lymph node metastases, poorer histologic differentiation and pathologic tumor status, a higher recurrence and distant metastases rate, and a higher rate of vascular and neural invasion.

Tumor necrosis is distinguished by the presence of dead cells with preserved overall tissue architecture. Rapid tumor growing may lead to relative hypoperfusion of the tumor's central area and hypoxia and nutritional deficiencies, which can cause tumor necrosis.^30^, ^39^ The cellular contents of necrotic tumor cells are released, flooding the region with pro‐inflammatory and tumor‐promoting cytokines which then attract immunological and inflammatory cells.^40^ Cell debris generated from necrotic cells can initiate an inflammatory response, and remodel the phenotypes of immune cells and tumor microenvironment to promote immune evasion.^41^, ^42^ In addition, tumor necrosis can cause abnormal structure and function of neovascularization in tumors, leading to abnormal leakiness of vasculature, thereby providing new routes for tumor metastasis. Hypoxia can increase the expression of hypoxia‐inducible factor 1‐α and induce epithelial‐mesenchymal transition to promote tumor cell dissemination.^43^ Furthermore, hypoxia may cause genome instability and change DNA damage pathways, which can enhance resistance to treatment.^42^ All in all, tumor necrosis has been considered a marker of tumor aggressiveness, and indicative of poor prognosis.

Several key limitations are present in this meta‐analysis study. First, due to the small number of included studies there may be a large effect publication bias and the Egger's and Begg's tests have low efficacies in terms of accurately reflecting the true publication bias when the number of included studies is less than 10. This study included only 10 articles; therefore, the results of our publication bias analysis should be taken with some caution. Second, owing to the lack of prospective studies, all the enrolled articles were retrospective cohorts, which is likely to have introduced inherent potential bias. Third, there may be some risks of selection bias. Our study pulled worldwide population‐based cohort studies with relatively large numbers of patients,^30^, ^31^ and these studies were assigned great weight. As a result, the pooled effect sizes tended to be consistent with the results of these population‐based cohort studies. Additionally, only published articles written in English were included, which may also have resulted in selection bias. Fourth, the criteria for detecting the presence of histopathological tumor necrosis, classifying the tumor size and determining the tumor necrosis degree were different in the different studies pulled, and we considered the DSS to be equivalent to OS to when we performed our subgroup analyses, which may have led to heterogeneity and inaccurate results. Finally, this study extracted the HRs and 95% CIs from several included studies utilizing Engauge Digitizer software, which may also have introduced bias.

## 5 CONCLUSIONS

This meta‐analysis and systematic review indicated that histopathological tumor necrosis is correlated with worse OS, DSS, and RFS trends in HBP cancers. Furthermore, tumor necrosis is not an isolated histopathological characteristic, and it is strongly linked to many other aggressive pathological features. In conclusion, tumor necrosis has the potential to represent a prospective prognostic factor in patients with HBP cancers. However, owing to the restrictions of the current articles, multicenter and high‐quality prospective researches with sufficient follow‐up times and larger numbers of patients are required to confirm these findings and draw more definitive conclusions.

## AUTHOR CONTRIBUTIONS

**Siqi Yang:** Data curation (equal); investigation (equal); writing – original draft (equal). **Haijie Hu:** Conceptualization (equal); funding acquisition (equal); writing – original draft (equal); writing – review and editing (equal). **Yafei Hu:** Data curation (supporting). **Yushi Dai:** Data curation (supporting). **Ruiqi Zou:** Data curation (supporting); visualization (supporting). **Tianrun Lv:** Data curation (supporting). **Fuyu Li:** Conceptualization (equal); funding acquisition (lead); project administration (equal); writing – review and editing (equal).

## FUNDING INFORMATION

This work was supported by 1.3.5 project for disciplines of excellence, West China Hospital, Sichuan University (ZYJC21046); 1.3.5 project for disciplines of excellence‐Clinical Research Incubation Project, West China Hospital, Sichuan University (2021HXFH001); Natural Science Foundation of Sichuan Province (2022NSFSC0806); National Natural Science Foundation of China for Young Scientists Fund (82203782); Sichuan Science and Technology Program (2021YFS0100); The fellowship of China Postdoctoral Science Foundation (2021M692277); Sichuan University‐Zigong School‐local Cooperation project (2021CDZG‐23); Science and Technology project of the Health planning committee of Sichuan (21PJ046); Post‐Doctor Research Project, West China Hospital, Sichuan University (2020HXBH127).

## CONFLICT OF INTEREST STATEMENT

The authors have no conflicts of interest to disclose.

## ETHICS APPROVAL AND CONSENT TO PARTICIPATE

Not applicable.

## CONSENT FOR PUBLICATION

Not applicable.

## Supporting information


Appendix S1

Appendix S2

## Data Availability

Not applicable.
