# Supplementary material for: Is tumor necrosis a clinical prognostic factor in hepato‐biliary‐pancreatic cancers? A systematic review and meta‐analysis
Source: Cancer Med. 2023 Mar 23;12(10):11166–76. doi: 10.1002/cam4.5742 (PMC10242339; doi:10.1002/cam4.5742)
Supplement: Supplementary file 1 — Appendix S1 [file CAM4-12-11166-s002.docx]

**Search strategies**

1. **Web of science queries**

#1 **TS=(tumor necrosis) OR ALL=(tumor necrosis) OR (ALL=(tumor) AND ALL=(necrosis))**

**#2 TS=(Liver Neoplasms) OR TS=(Hepatic Neoplasm) OR TS=(Cancer of Liver) OR TS=(Hepatocellular Cancer) OR TS=(Hepatic Cancer) OR TS=(Liver Cancer) OR TS=(Hepatocellular Carcinoma) OR TS=(Liver Cell Carcinoma) OR TS=(Adult Liver Cancer) OR TS=(Hepatoma)**

**#3 TS=(Biliary Tract Neoplasms) OR TS=(Biliary Tract Cancer) OR TS=(Cancer of Biliary Tract) OR TS=(Cholangiocarcinoma) OR TS=(Cholangiocellular Carcinoma) OR TS=(Extrahepatic Cholangiocarcinoma) OR TS=(Intrahepatic Cholangiocarcinoma)**

**#4 TS=(Pancreatic Neoplasms) OR TS=(Pancreas Neoplasm) OR TS=(Cancer of Pancreas) OR TS=(Pancreas Cancer) OR TS=(Pancreatic Cancer) OR TS=(Cancer of the Pancreas)**

**#5 #2 OR #3 OR #4**

**#6 TS=(prognosis) OR TS=(Survival) OR TS=(survival analysis) OR TS=(mortality) OR TS=(death rate*) OR TS=(cox regression) OR TS=(proportional hazard)**

**#7 #1 AND #5 AND #6**

1. **PubMed queries**

**(("tumor necrosis"[All Fields] OR ("tumor"[All Fields] AND "necrosis"[All Fields])) AND ("biliary tract neoplasms"[MeSH Terms] OR ("biliary"[All Fields] AND "tract"[All Fields] AND "neoplasms"[All Fields]) OR "biliary tract neoplasms"[All Fields] OR ("biliary"[All Fields] AND "cancer"[All Fields]) OR "biliary cancer"[All Fields] OR "Cholangiocarcinoma"[MeSH Terms] OR ("Cholangiocellular"[All Fields] AND "Carcinoma"[All Fields]) OR "Cholangiocellular Carcinoma"[All Fields] OR ("Extrahepatic"[All Fields] AND "Cholangiocarcinoma"[All Fields]) OR "Extrahepatic Cholangiocarcinoma"[All Fields] OR "Intrahepatic Cholangiocarcinoma"[All Fields] OR ("Intrahepatic"[All Fields] AND "Cholangiocarcinoma"[All Fields]) OR ("liver neoplasms"[MeSH Terms] OR ("liver"[All Fields] AND "neoplasms"[All Fields]) OR "liver neoplasms"[All Fields] OR ("liver"[All Fields] AND "cancer"[All Fields]) OR "liver cancer"[All Fields]) OR ("pancreatic neoplasms"[MeSH Terms] OR ("pancreatic"[All Fields] AND "neoplasms"[All Fields]) OR "pancreatic neoplasms"[All Fields] OR ("pancreatic"[All Fields] AND "cancer"[All Fields]) OR "pancreatic cancer"[All Fields])) AND ("prognosis"[MeSH Terms] OR "Survival"[MeSH Terms] OR "survival analysis"[MeSH Terms] OR "mortality"[MeSH Terms] OR "Survival"[Title/Abstract] OR "mortalit*"[Title/Abstract] OR "death rate*"[Title/Abstract] OR "cox regression"[Title/Abstract] OR "proportional hazard"[Title/Abstract]))**

1. **EMBASE**

**#7 #2 AND #5 AND #6**

**#6 #1 OR #3 OR #4**

**#5 'prognosis'/exp OR 'survival'/exp OR 'survival analysis'/exp OR 'mortality'/exp OR 'cox regression':ab,ti OR 'proportional hazard':ab,ti OR 'death rate*':ab,ti**

**#4 'pancreas tumor'/exp OR 'pancreatic neoplasm':ab,ti OR 'pancreas neoplasm':ab,ti OR 'pancreas cancer':ab,ti OR 'pancreatic cancer':ab,ti**

**#3 'liver tumor'/exp OR 'hepatic neoplasm':ab,ti OR 'cancer of liver':ab,ti OR 'hepatocellular cancer':ab,ti OR 'hepatic cancer':ab,ti OR 'cancer of the liver':ab,ti OR 'liver cell carcinoma'/exp OR 'hepatocellular carcinoma':ab,ti OR 'adult liver cancer':ab,ti OR 'liver cell carcinoma':ab,ti OR 'hepatoma':ab,ti**

**#2 'tumor necrosis'/exp**

**#1 'biliary tract tumor'/exp OR 'biliary tract tumor' OR 'biliary tract neoplasm':ab,ti OR 'cancer of biliary tract':ab,ti OR 'bile duct cancer':ab,ti OR 'cholangiocarcinoma':ab,ti OR 'cholangiocellular carcinoma':ab,ti OR 'extrahepatic cholangiocarcinoma':ab,ti OR 'intrahepatic cholangiocarcinoma':ab,ti**

1. **Cochrane library**

#1 (tumor necrosis) (Word variations have been searched) 14289

#2 (tumor) (Word variations have been searched) 88045

#3 (necrosis) (Word variations have been searched) 19390

#4 (#1 AND (#2 OR #3)) 14203

#5 MeSH descriptor: [Liver Neoplasms] explode all trees 3374

#6 (liver cancer) (Word variations have been searched) 14778

#7 (liver) 70620

#8 (cancer) 213363

#9 #5 OR #6 OR (#7 AND #8) 16009

#10 MeSH descriptor: [Biliary Tract Neoplasms] explode all trees 517

#11 (biliary) 7473

#12 (tract) 46053

#13 (neoplasms) 77566

#14 (biliary tract neoplasms) 385

#15 #10 OR (#11 AND #12 AND #13) OR #14 640

#16 (biliary cancer) 1866

#17 (biliary) 7473

#18 cancer 213363

#19 #10 OR (#11 AND #12 AND #13) OR #14 OR #16 OR (#17 AND #18) 2157

#20 MeSH descriptor: [Pancreatic Neoplasms] explode all trees 2035

#21 (pancreatic) 12370

#22 (neoplasms) 77566

#23 (pancreatic neoplasms) 2488

#24 (pancreatic) 12370

#25 (cancer) 213363

#26 (pancreatic cancer) 5809

#27 #20 OR (#21 AND #22) OR #23 OR (#24 AND #25) OR #26 6344

#28 MeSH descriptor: [Prognosis] explode all trees 170193

#29 MeSH descriptor: [Survival] explode all trees 134

#30 MeSH descriptor: [Survival Analysis] explode all trees 21961

#31 MeSH descriptor: [Mortality] explode all trees 14102

#32 (Survival):ti,ab,kw 123166

#33 (death rate*):ti,ab,kw 34514

#34 (cox regression):ti,ab,kw 9854

#35 (proportional hazard):ti,ab,kw 8413

#36 #28 OR #29 OR #30 OR #31 OR #32 OR #33 OR #34 OR #35 293240

#37 #9 OR #19 OR #27 22202

#38 #4 AND #36 AND #37 209

#39 MeSH descriptor: [Cholangiocarcinoma] explode all trees 266

#40 (Cholangiocellular Carcinoma) (Word variations have been searched) 23

#41 (Cholangiocellular) (Word variations have been searched) 25

#42 (Carcinoma) (Word variations have been searched) 47486

#43 (Extrahepatic) (Word variations have been searched) 1356

#44 (Cholangiocarcinoma) (Word variations have been searched) 924

#45 (Extrahepatic Cholangiocarcinoma) (Word variations have been searched) 188

#46 (Intrahepatic Cholangiocarcinoma) (Word variations have been searched) 333

#47 (Intrahepatic) (Word variations have been searched) 2099

#48 #39 OR #40 OR (#41 AND #42) OR #45 OR (#43 AND #44) OR #45 OR (#47 AND #44) 576

#49 #19 OR #48 2413

#50 #9 OR #49 OR #27 22366

#51 #4 AND #50 AND #36 209

**Table S2. The details of enrolled studies**

| Study | T stage  T1-2/T3-4 | Resection margin  R0/R1 | Differentiation  Well, Moderate/Poor | adjuvent therapy | OS or DSS HR/OR/RR (95% CI) P-value | | RFS or DFS HR/OR/RR (95% CI) P-value | |
| --- | --- | --- | --- | --- | --- | --- | --- | --- |
|  |  |  |  |  | univariate analysis | multivariate analysis | univariate analysis | multivariate analysis |
| Mitsunaga et al. (2005) | 7/94 | 83/18 | 52/49 | N/A | N/A | 2.6 (1.5-5.0) 0.004 | N/A | 3.8 (2.0-7.5) <0.001 |
| Hiraoka et al. (2010) | 9/339 | 249/100 | 271/77 | Chemotherapy and radiotherapy | 2.196 (1.659–2.905)  <0.001 | 2.238 (1.686–2.971)  <0.001 | 2.007 (1.531–2.630)  <0.0001 | 1.853 (1.407–2.440) <0.001 |
| Atanasov et al. (2017) | 35/53 | N/A | 60/28 | N/A | N/A | 0.244 (0.100-0.596) 0.002 ^a^ | N/A | N/A |
| Atanasov et al. (2017) | N/A | 37/10 | N/A | N/A | N/A | N/A | N/A | 0.369 (0.158-0.859) 0.021 ^a^ |
| Atanasov et al. (2019) | 29/29 | 49/9 | 11/47 ^b^ | N/A | N/A | N/A | N/A | N/A |
| Kudo et al. (2020) | 72/149 ^c^ | N/A | N/A | Chemotherapy | 4.12 (2.54-6.97) <0.001 | 3.16 (1.91-5.44) <0.001 | N/A | N/A |
| Ling et al.  (2020) | N/A | N/A | 264/71 | N/A | 2.821 (1.643-4.842) <0.0001 | 2.208(1.272-3.833) 0.005 | N/A | N/A |
| Wei et al.  (2021) | 769/109 | N/A | 672/210 | N/A | N/A | N/A | N/A | N/A |
| Tsilimigras et al. (2022) | 659/98 | 618/133 | 525/198 | Chemotherapy | N/A | N/A | N/A | N/A |
| Kuo et al. (2023) | 660/96 | N/A | 719/32 | N/A | 2.421 (1.776-3.300) <0.01 | 1.956 (1.409-2.716) <0.001 | 1.714 (1.325-2.216) <0.001 | 1.422 (1.114-1.816) 0.005 |

^a^: The data was obtained by comparing the absent tumor necrosis and present necrosis.

^b^: The data was shown in well/moderate and poor.

^C^: The data was shown in T1/T2

**Table S2. Quality assessment of cohort studies included in this meta- analysis**

| **Study** | **Representativeness of the exposed cohort** | **Selection of the unexposed cohort** | **Ascertainment of exposure** | **Outcome of interest not present at start of study** | **Control for important factor or additional factor** | **Outcome assessment** | **Follow-up long enough for outcomes to occur** | **Adequacy of follow-up of cohort** | **Total quality scores** |
| --- | --- | --- | --- | --- | --- | --- | --- | --- | --- |
| Mitsunaga et al. 2005 | ★ | ★ | ★ | ★ | ★ | ★ | — | ★ | 7 |
| Hiraoka et al. 2010 | ★ | ★ | ★ | ★ | ★ | ★ | ★ | ★ | 8 |
| Atanasov et al. 2017 | ★ | ★ | ★ | ★ | ★ | ★ | ★ | ★ | 8 |
| Atanasov et al. 2017 | ★ | ★ | — | ★ | — | ★ | ★ | ★ | 6 |
| Atanasov et al. 2019 | ★ | ★ | ★ | ★ | ★ | ★ | ★ | ★ | 8 |
| Kudo et al. 2020 | ★ | ★ | ★ | ★ | ★ | ★ | — | ★ | 7 |
| Ling et al. 2020 | ★ | ★ | ★ | ★ | ★ | ★ | — | ★ | 7 |
| Wei et al. 2021 | ★ | ★ | ★ | ★ | ★ | ★ | ★ | ★ | 8 |
| Tsilimigras et al. 2022 | ★ | ★ | ★ | ★ | ★ | ★ | ★ | ★ | 8 |
| Kuo et al. 2023 | ★ | ★ | ★ | ★ | ★ | ★ | — | ★ | 7 |


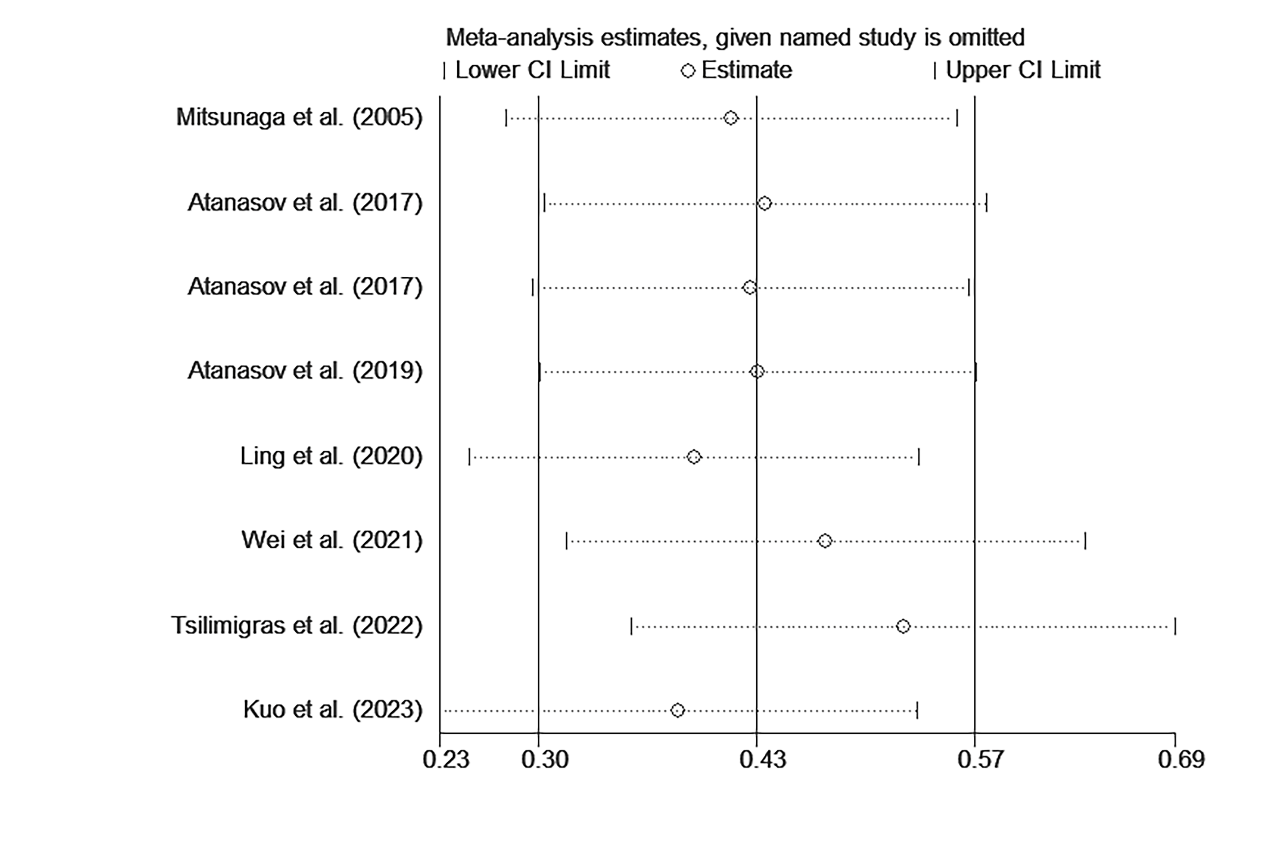


**Figure S1**. The sensitivity analysis of OS.


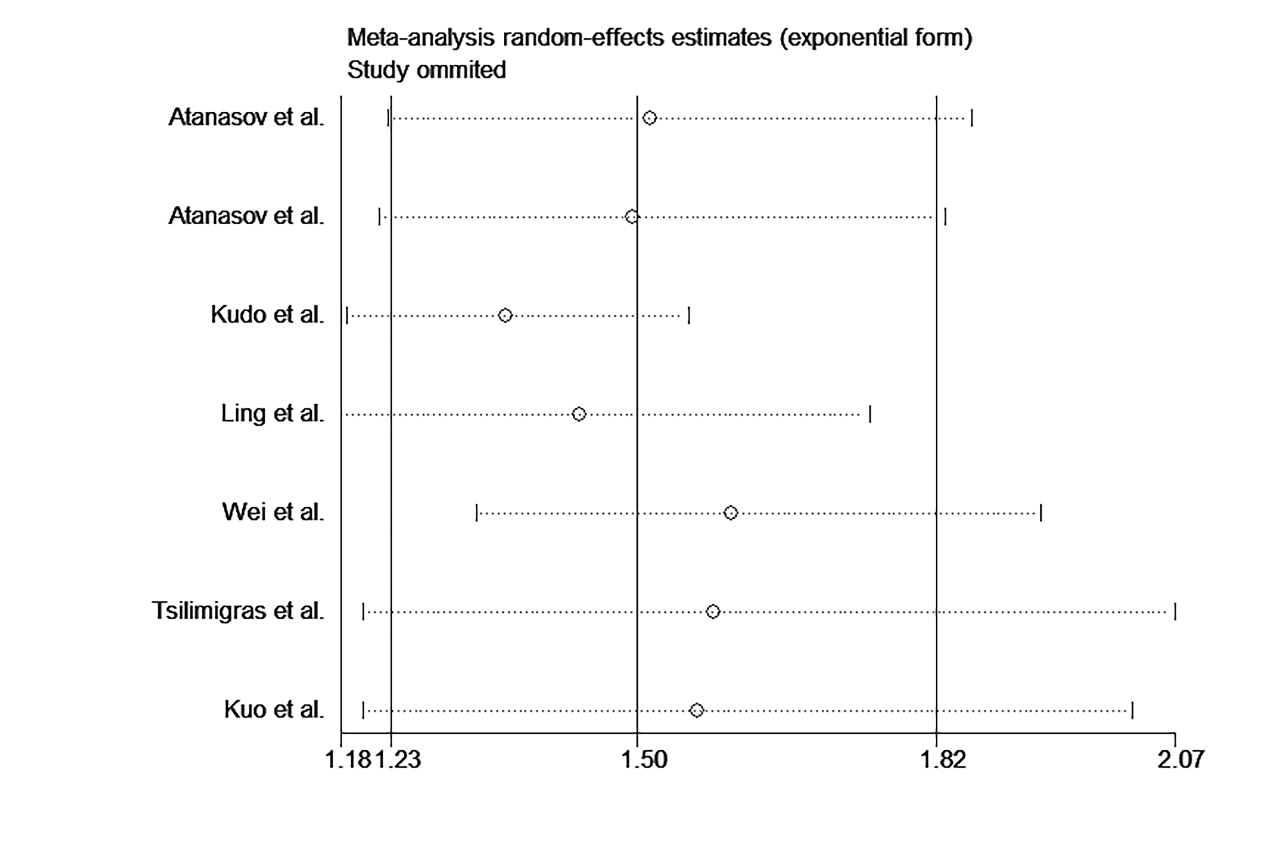


**Figure S2**. The sensitivity analysis of RFS.

**
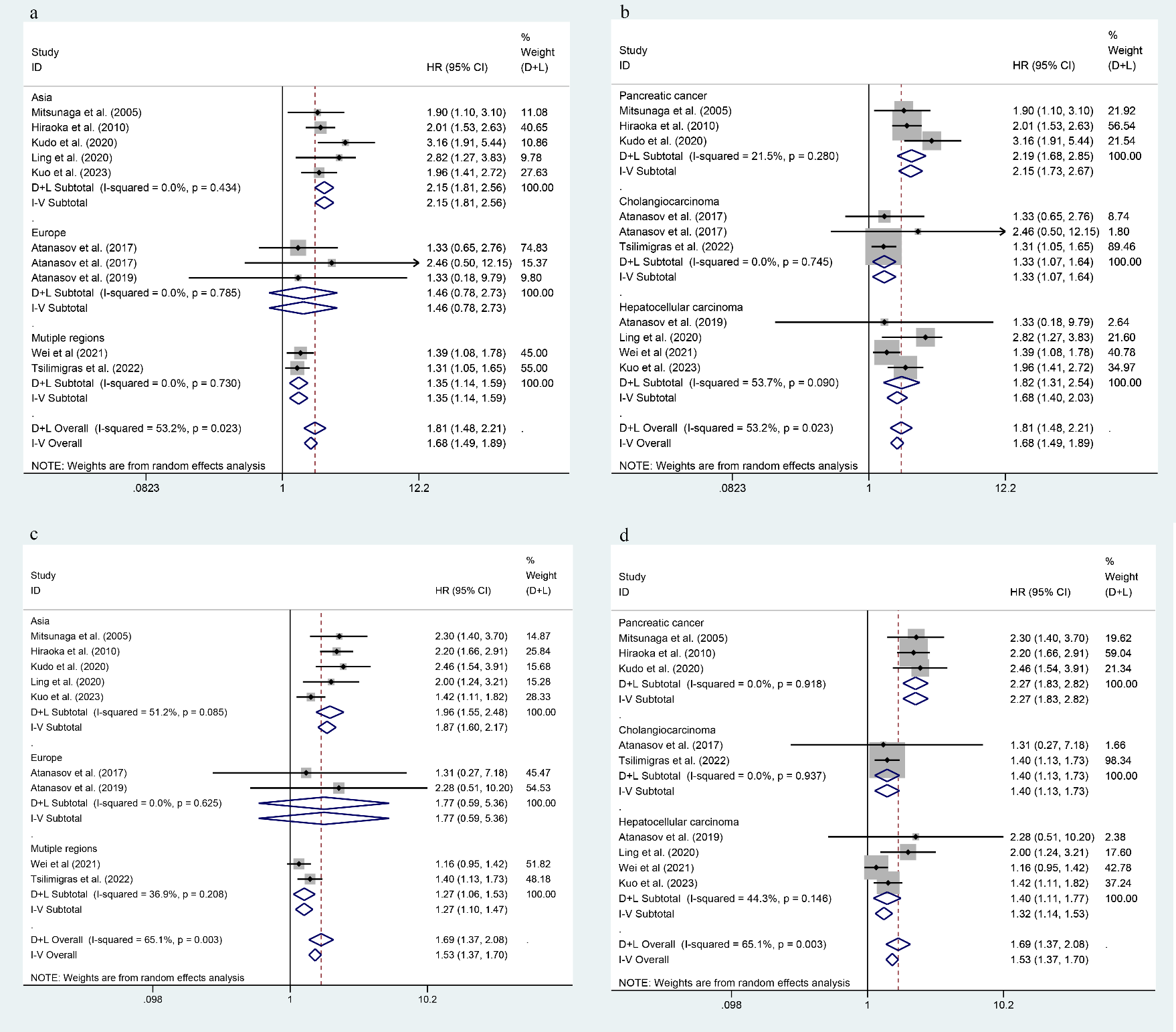
Fig.S3**. Subgroup analysis for the association between tumor necrosis and survival outcomes of HPB cancers. a, region subgroup for OS; b, cancer type subgroup for OS; c, region subgroup for RFS; d, cancer type subgroup for RFS.





**Fig.S4**. Meta-analysis of the association between tumor necrosis and tumor size.





**Fig.S5.** Meta-analysis of the association between tumor necrosis and tumor nodule number.





**Fig.S6.** Meta-analysis of the association between tumor necrosis and lymph node metastases.





**Fig.S7**. Meta-analysis of the association between tumor necrosis and vascular invasion.





**Fig.S8.** Meta-analysis of the association between tumor necrosis and neural invasion.

**

**

**Fig.S9.** Meta-analysis of the association between tumor necrosis and pathologic tumor status.





**Fig.S10.** Meta-analysis of the association between tumor necrosis and histologic differentiation.

**

**

**Fig.S11.** Meta-analysis of the association between tumor necrosis and R0 resection.





**Fig.S12.** Meta-analysis of the association between tumor necrosis and recurrence.





**Fig.S13.** Meta-analysis of the association between tumor necrosis and distant metastases.





**Fig.S14.** Funnel plot with P_values of Begg’s and Egger’s test.
