# Supplementary material for: Is tumor necrosis a clinical prognostic factor in hepato‐biliary‐pancreatic cancers? A systematic review and meta‐analysis
Source: Cancer Med. 2023 Mar 23;12(10):11166–76. doi: 10.1002/cam4.5742 (PMC10242339; doi:10.1002/cam4.5742)
Supplement: Supplementary file 2 — Appendix S2 [file CAM4-12-11166-s001.docx]

**Table S1. The details of enrolled studies**

| Study | T stage  T1-2/T3-4 | Resection margin  R0/R1 | Differentiation  Well, Moderate/Poor | adjuvent therapy | OS or DSS HR/OR/RR (95% CI) P-value | | RFS or DFS HR/OR/RR (95% CI) P-value | |
| --- | --- | --- | --- | --- | --- | --- | --- | --- |
|  |  |  |  |  | univariate analysis | multivariate analysis | univariate analysis | multivariate analysis |
| Mitsunaga et al. (2005) | 7/94 | 83/18 | 52/49 | N/A | N/A | 2.6 (1.5-5.0) 0.004 | N/A | 3.8 (2.0-7.5) <0.001 |
| Hiraoka et al. (2010) | 9/339 | 249/100 | 271/77 | Chemotherapy and radiotherapy | 2.196 (1.659–2.905)  <0.001 | 2.238 (1.686–2.971)  <0.001 | 2.007 (1.531–2.630)  <0.0001 | 1.853 (1.407–2.440) <0.001 |
| Atanasov et al. (2017) | 35/53 | N/A | 60/28 | N/A | N/A | 0.244 (0.100-0.596) 0.002 ^a^ | N/A | N/A |
| Atanasov et al. (2017) | N/A | 37/10 | N/A | N/A | N/A | N/A | N/A | 0.369 (0.158-0.859) 0.021 ^a^ |
| Atanasov et al. (2019) | 29/29 | 49/9 | 11/47 ^b^ | N/A | N/A | N/A | N/A | N/A |
| Kudo et al. (2020) | 72/149 ^c^ | N/A | N/A | Chemotherapy | 4.12 (2.54-6.97) <0.001 | 3.16 (1.91-5.44) <0.001 | N/A | N/A |
| Ling et al.  (2020) | N/A | N/A | 264/71 | N/A | 2.821 (1.643-4.842) <0.0001 | 2.208(1.272-3.833) 0.005 | N/A | N/A |
| Wei et al.  (2021) | 769/109 | N/A | 672/210 | N/A | N/A | N/A | N/A | N/A |
| Tsilimigras et al. (2022) | 659/98 | 618/133 | 525/198 | Chemotherapy | N/A | N/A | N/A | N/A |
| Kuo et al. (2023) | 660/96 | N/A | 719/32 | N/A | 2.421 (1.776-3.300) <0.01 | 1.956 (1.409-2.716) <0.001 | 1.714 (1.325-2.216) <0.001 | 1.422 (1.114-1.816) 0.005 |

^a^: The data was obtained by comparing the absent tumor necrosis and present necrosis.

^b^: The data was shown in well/moderate and poor.

^C^: The data was shown in T1/T2

**Table S2. Quality assessment of cohort studies included in this meta- analysis**

| **Study** | **Representativeness of the exposed cohort** | **Selection of the unexposed cohort** | **Ascertainment of exposure** | **Outcome of interest not present at start of study** | **Control for important factor or additional factor** | **Outcome assessment** | **Follow-up long enough for outcomes to occur** | **Adequacy of follow-up of cohort** | **Total quality scores** |
| --- | --- | --- | --- | --- | --- | --- | --- | --- | --- |
| Mitsunaga et al. 2005 | ★ | ★ | ★ | ★ | ★ | ★ | — | ★ | 7 |
| Hiraoka et al. 2010 | ★ | ★ | ★ | ★ | ★ | ★ | ★ | ★ | 8 |
| Atanasov et al. 2017 | ★ | ★ | ★ | ★ | ★ | ★ | ★ | ★ | 8 |
| Atanasov et al. 2017 | ★ | ★ | — | ★ | — | ★ | ★ | ★ | 6 |
| Atanasov et al. 2019 | ★ | ★ | ★ | ★ | ★ | ★ | ★ | ★ | 8 |
| Kudo et al. 2020 | ★ | ★ | ★ | ★ | ★ | ★ | — | ★ | 7 |
| Ling et al. 2020 | ★ | ★ | ★ | ★ | ★ | ★ | — | ★ | 7 |
| Wei et al. 2021 | ★ | ★ | ★ | ★ | ★ | ★ | ★ | ★ | 8 |
| Tsilimigras et al. 2022 | ★ | ★ | ★ | ★ | ★ | ★ | ★ | ★ | 8 |
| Kuo et al. 2023 | ★ | ★ | ★ | ★ | ★ | ★ | — | ★ | 7 |


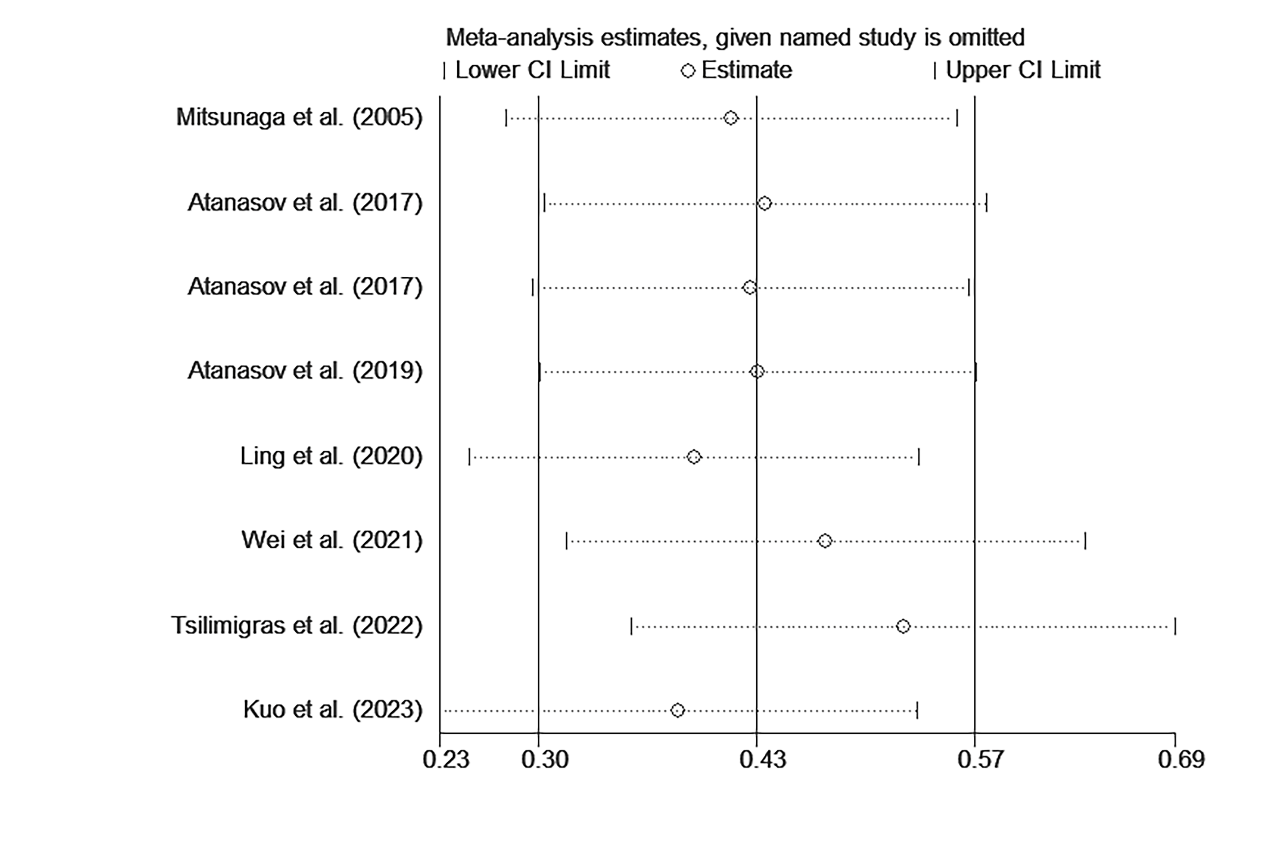


**Figure S1**. The sensitivity analysis of OS.


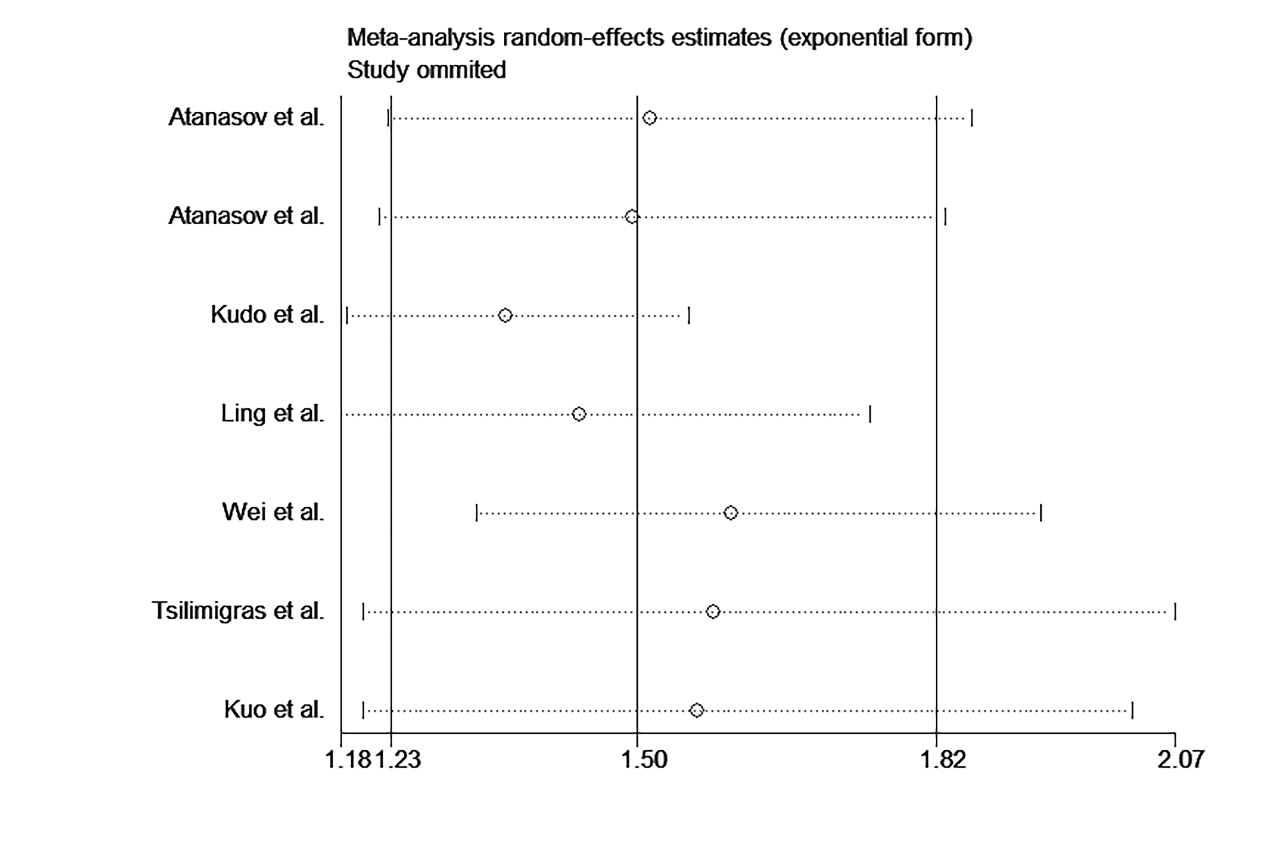


**Figure S2**. The sensitivity analysis of RFS.

**
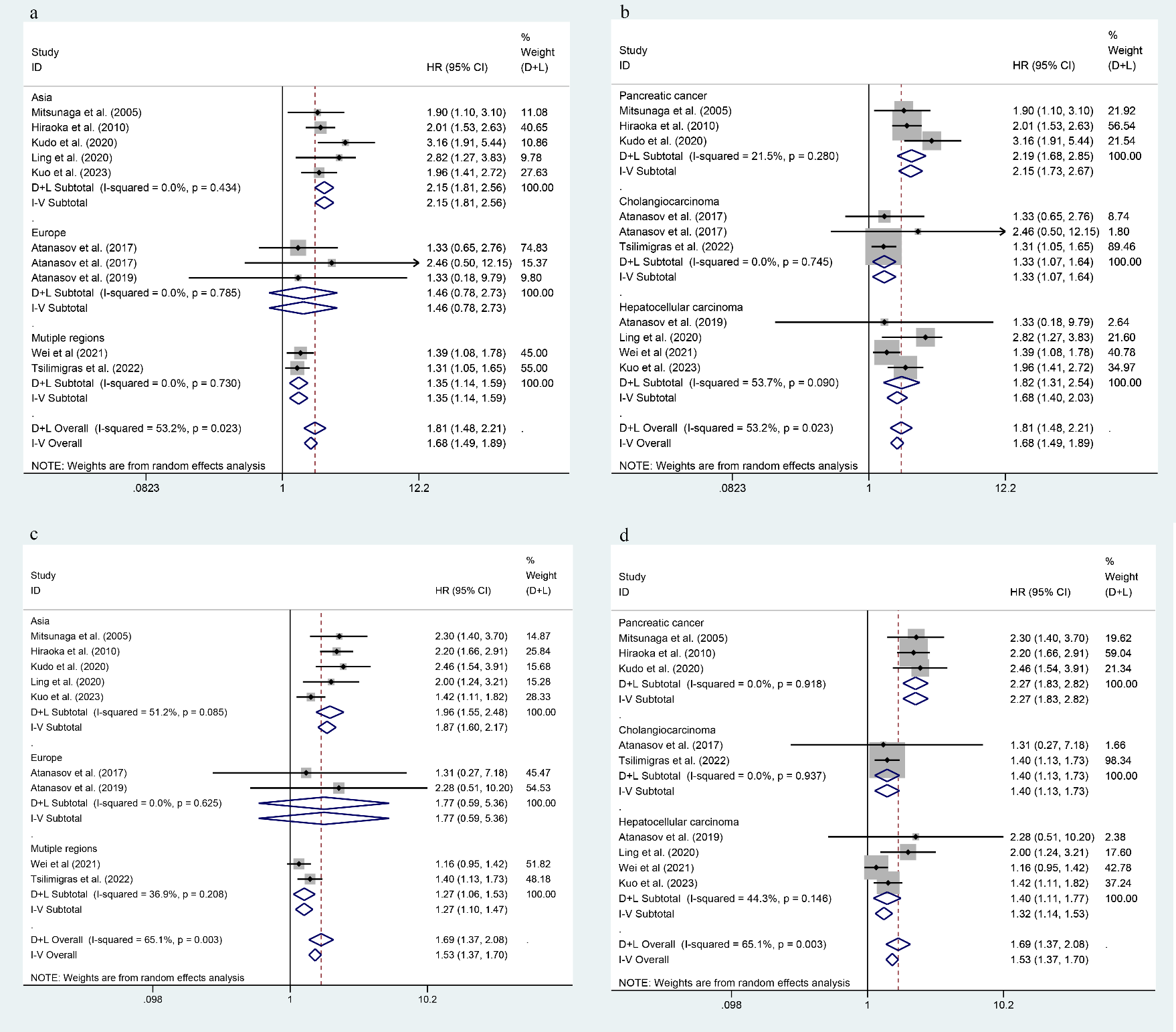
Fig.S3**. Subgroup analysis for the association between tumor necrosis and survival outcomes of HPB cancers. a, region subgroup for OS; b, cancer type subgroup for OS; c, region subgroup for RFS; d, cancer type subgroup for RFS.





**Fig.S4**. Meta-analysis of the association between tumor necrosis and tumor size.





**Fig.S5.** Meta-analysis of the association between tumor necrosis and tumor nodule number.





**Fig.S6.** Meta-analysis of the association between tumor necrosis and lymph node metastases.





**Fig.S7**. Meta-analysis of the association between tumor necrosis and vascular invasion.





**Fig.S8.** Meta-analysis of the association between tumor necrosis and neural invasion.

**

**

**Fig.S9.** Meta-analysis of the association between tumor necrosis and pathologic tumor status.





**Fig.S10.** Meta-analysis of the association between tumor necrosis and histologic differentiation.

**

**

**Fig.S11.** Meta-analysis of the association between tumor necrosis and R0 resection.





**Fig.S12.** Meta-analysis of the association between tumor necrosis and recurrence.





**Fig.S13.** Meta-analysis of the association between tumor necrosis and distant metastases.





**Fig.S14.** Funnel plot with P_values of Begg’s and Egger’s test.
